# Molecular targeting of the meningioma cell signaling circuit reveals drug vulnerabilities including synergy between sulforaphane and focal adhesion kinase inhibition

**DOI:** 10.1038/s44276-026-00204-2

**Published:** 2026-04-15

**Authors:** Michelle C. Schwab, Yasmin Kocas, Sarah Gendreizig, Eric Goulin Lippi Fernandes, Tunc F. Ersoy, Roland Coras, Lars Uwe Scholtz, Holger Sudhoff, Ingo Todt, Matthias Simon, Matthias Schürmann, Felix Oppel

**Affiliations:** 1 Department of Otolaryngology, Head and Neck Surgery, Campus Klinikum Bielefeld Mitte, University Hospital OWL of Bielefeld University, Bielefeld, Germany; 2 EUREGIO BioMedtech Center, University of Applied Sciences Münster, Steinfurt, Germany; 3 Department of Neurosurgery, Campus Evangelisches Krankenhaus Bethel, University Hospital OWL of Bielefeld University, Bielefeld, Germany; 4 Department of Neurosurgery, Isala Klinieken, Zwolle, The Netherlands; 5 Department of Neuropathology, FAU Erlangen-Nürnberg, University Hospital Erlangen, Erlangen, Germany

## Abstract

**Background:**

Meningiomas represent a common type of brain tumours and arise from the arachnoid cap cells which line the dural coverings of the brain. These predominantly benign tumours cause strong morbidity due to their intracranial location. Due to a lack of established pharmacological treatment approaches, surgery and radiotherapy remain the standard in clinical management of these tumours.

**Methods:**

Here, we created a primary meningioma cell culture model and tested molecular compounds targeting key tumour-driving signalling pathways using cell viability assays and isobologram analysis.

**Results:**

We found significant drug vulnerabilities within the meningioma-driving cell signalling network. Effective drugs included metformin, inhibitors of STAT3 and focal adhesion kinase (FAK), and the anti-inflammatory phytochemical sulforaphane, known as NRF2 pathway inducer and NFκB pathway inhibitor. Interestingly, FAK inhibitor Y15 and sulforaphane showed synergistic activity across cells of distinct meningioma patients, indicating this combination as a potential therapy approach.

**Conclusions:**

Overall, we present a molecular targeting strategy for meningiomas that could pave the way for less invasive clinical management of these tumours and, as a result, help reduce patient mortality and morbidity. Metformin and sulforaphane both have FDA and EU pharmaceutical approval and thus could be repurposed promptly to establish a new meningioma therapy regimen.

## Introduction

Meningioma is the most common intracranial tumour type, representing about 37.6% of these neoplasms, having an incidence of 8.33 cases per 100,000 person-years [1–3]. The tumour cells arise from the arachnoid cap cells and dural border cells of the meninges [4, 5]. Meningiomas can occur at any age, but most likely at the age range of 45–55 years [6]. Due to their typically slow growth, neurologic deficits and pain develop gradually over the course of the disease accompanied by symptoms depending on tumour location [1]. The majority of meningiomas are benign, but about 20% show more aggressive behaviour. Up to 80% of all meningioma are categorised as WHO grade I with 9 subtypes, up to 20% as grade II with 3 subtypes and 1–3% as grade III with 3 subtypes [1, 7]. Risk factors for the development of meningiomas include ionising radiation to the skull, genetic alterations and sex hormones, as the disease is more common in females [1]. Surgery is the standard treatment, but in some cases radiosurgery is preferred over conventional surgical resections [1, 8]. Given the higher recurrence risk of grade II (29–52%) and grade III (50–94%) meningiomas [7, 9], radiation therapy is commonly employed as an adjunct treatment for refractory tumours as well as for inoperable grade I tumours, though with variable effectiveness [10].

The most frequently observed genetic alterations in meningiomas affect the *NF2* gene, located on chromosome 22 [11]. The *NF2* gene encodes merlin, a cytoskeletal linker protein that is part of the ERM (Ezrin-Radixin-Moesin) family [12]. Besides its role in membrane stability, regulation of cell motility, regulation of cytoskeleton remodelling and cell-cell contact inhibition [13, 14], it also acts as a tumour suppressor by regulating tumour growth-associated pathways, including NFκB, JAK/STAT, JNK, mTOR/AKT and many others [14–18]. As this gene is mutated in about 60% of all meningiomas, it may be a promising target for medical therapy approaches [11].

A substantial number of patients eventually fail surgery and radiotherapy and these treatments carry significant complication rates [19–21]. There is no established pharmacotherapy approach for meningiomas. Hence, there is an urgent need to develop new treatment options. Following the example set in other fields of neuro-oncology [22], searching for a molecular targeted therapy paradigm may be promising. In this study, we therefore investigated the impact of inhibitors and substrates that interfere with key signalling pathways in three primary meningioma cell cultures. Sulforaphane, a bioactive compound found in cruciferous vegetables, is known for its anti-inflammatory properties [23] and to interfere with tumour-initiating cells [24]. We demonstrate that sulforaphane synergistically enhances the effect of FAK inhibition in targeting meningioma cells, suggesting its potential as a valuable component in novel pharmacotherapeutic strategies for this tumour.

## Methods

### Human materials

The primary meningioma cell cultures created from surgically removed tumour tissue, purified and cultured as described below. Primary nasal fibroblasts were derived from a previous study [25]. Ethics approval and patients‘ consent were obtained as declared below.

### Tissue purification

Meningioma tissue samples were transported in sterile 1x Phosphate-buffered Saline (Capricorn Scientific, Germany, # PBS-10XA) (PBS) on ice and then placed in a sterile Petri dish. Blood vessels were removed from the tissue, a piece of the tissue was fixed in 4% paraformaldehyde (PFA) over night and the remaining tissue was then sliced into 2 mm pieces using a scalpel in 100× Penicillin/Streptomycin (Capricorn Scientific, Germany, # PS-B) and 100x Amphotericin B Solution (Capricron Scientific, Germany, # AMP-B). The tissue pieces were transferred into a 50 mL falcon tube with 30 mL 1× PBS and gently inverted a few times. The falcon tube was then centrifuged at 1000 rpm for 5 min (min), 4 °C, using a large-volume cell culture centrifuge (Hermle, Germany, model Z446K). This washing step was performed three times. The pieces were then incubated in 10 mL DMEM cultivation medium containing 1.28 mg/mL collagenase type I (Sigma-Aldrich, Germany, # C0130) and 0.5 mg/mL hyaluronidase type I-S (Sigma-Aldrich, Germany, # H3506) for 18 h (h) at 37 °C (modified from Dilwali et al. 26). The pieces were washed with 1× PBS. For cell counting cells were staining with a trypan blue solution in a 1:1 dilution and then counted with a Neubauer chamber. 5 million viable cells were seeded in a T75 flask.

### Cell culture cultivation

The cells were cultivated in T75 flask in DMEM medium (Capricorn Scientific, Germany, # DMEM-HXA) containing 10% FCS (Capricorn Scientific, South America, # FCS-62A), 1% Penicillin/Streptomycin (Capricorn Scientific, Germany, # PS-B), 1% L-glutamine (Capricorn Scientific, Germany, # GLN-B) and 1% Amphotericin B Solution (Capricorn Scientific, Germany, # AMP-B) at 37 °C. The cultivation medium was replaced every 2–3 days.

At 70–90% confluency, the cells were detached and separated using 4 mL Accutase cell detachment solution (Capricorn Scientific, Germany, # ACC-1B) at 37 °C. 300,000 cells were reseeded in a T75 flask.

### Growth curve establishment

To establish a long-term growth curve, the cells were counted at every passage. The cell number at each passage was multiplied with the dilution factor of every previous reseeding step. Data was visualised using GraphPad Prism software (Graphpad Software Inc., San Diego, CA, United States).

### Fixation of cells

The cells were seeded in 1 mL DMEM medium in a 12-well plate on coverslips. For fixation, the cultivation medium was removed, and the cells were washed with 1 mL 1× PBS. Next, 1 mL 4% paraformaldehyde (PFA) was added to the cells for 20–25 min. Then the PFA was removed, the cells washed again with 1× PBS and stored in 1× PBS at 4 °C.

### Indirect immunoflourescence staining

Cells grown on cover slips in 12-wells were incubated with 1 mL permealization buffer (1× PBS containing 0.1% citrate and 0.1% Triton-X-100) for 10 min. The cells were then washed with blocking buffer composed of 1× PBS + 0.1% Bovine Serum Albumin (Capricorn Scientific, Germany, # BSA-1T). Then, the cells were stained for 10 min with a 1:500 DAPI solution in blocking buffer. The cells were then washed again. After that, 75 µL of a primary antibody dilution in blocking buffer was added to the coverslips and incubated at least 1.5 h. The antibodies and the respective dilutions are listed in Supplementary Table S1. After three washing steps, 75 µL secondary antibody dilution in blocking buffer was added to the cells and incubated for 1.5 h in the dark. Next, the cells were washed one time each with blocking buffer, PBS and water. The coverslip was then removed using tweezers and placed on a microscopy slide with 20 μl mounting medium (Santa Cruz Biotechnology, Dallas, TX, United States, # sc-516212). Samples were imaged using an LSM780 confocal microscope (Zeiss, Oberkochen, Germany) and ZEN software (Zeiss) or using a Keyence BZ-X800 microscope and Keyence software (BZ-X800 Viewer).

### Drug treatment experiments

Either 1500 (MG1 and MG3) or 1,000 cells (MG2) were seeded in 200 µL DMEM medium in 96-well plates. After 48 h, the cells were treated with compounds diluted in 100 µL medium per 96-well. The used drugs are listed in Supplementary Table S2. After 48 h a cell viability assay was performed using an MTS assay (Promega, Germany, #G3580). The medium was removed from the treated cells and 100 µL DMEM medium and 10 µL MTS solution were added. The cells were then incubated for 2 h at 37 °C. After that the absorbance was measured at 490 nm with 690 nm reference. The well plate was shaken before the measurement for 10 s and then rested for 10 s.

For screening experiments, drug concentrations were tested in three decimal powers to determine their effect range. These concentrations were chosen according to their IC_50_ values known from previous studies. Next, a dilution series of the identified drugs was tested to determine their IC_50_ in meningioma cell cultures using four technical replicates (*n* = 4). Obtained data were analysed employing GraphPad Prism software. The statistical significance was calculated using an unpaired Student’s *t* test with Welch´s correction. The minimum efficiency dose (MED) of the identified drugs was determined by using a One-way Brown-Forsythe and Welch ANOVA test with a Dunnett’s T3 multiple comparisons test in GraphPad Prism10.

### Combination drug treatment and isobologram analysis

The cooperation effect of two distinct drugs was determined as described previously [27, 28]. In brief, the combination rate (CR) of each pair of drugs was determined with the IC_50_ values of the respective single treatments, with IC_50_A being the IC_50_ of drug A, and IC_50_B being the IC_50_ of drug B.

$${{{\rm{CR}}}}={{{{\rm{IC}}}}}_{50}{{{\rm{A}}}}/{{{{\rm{IC}}}}}_{50}{{{\rm{B}}}}$$

Using the CR, the concentrations of drugs A and B in combination were determined by the formula:

$${C}_{{{{\rm{B}}}}}={C}_{{{{\rm{A}}}}}/{{\mathrm{CR}}}$$

A dilution series of the combined drugs was then tested in the primary meningioma cell cultures using four technical replicates (*n* = 4). With the concentrations of drugs A and B at the IC_50_ value in the single and in the combined treatment the combination index (CI) can then be determined.

$${{{\rm{CI}}}}={{{\rm{a}}}}/{{{\rm{A}}}}+{{{\rm{b}}}}/{{{\rm{B}}}}$$

With a and b being the IC_50_ concentrations of the drugs in combination treatment and A and B being the concentrations in single treatment. A CI < 1 shows drug synergy, CI = 1 additivity, and CI > 1 represents antagonism [28]. Isobologram plots were subsequently created by connecting the IC_50_ dose value from the single treatment experiment of each drug and adding the dot indicating the IC_50_ of both drugs in combination. Data was visualised using GraphPad Prism software.

### Histopathology analysis

FFPE tissue was cut into 3 μm thin sections with a microtome (Microm GmbH, Germany) and mounted on glass slides (Epredia SuperFrost Plus Adhesion Microscope Slides, USA). Immunohistochemical staining was performed utilising an automated staining system (Ventana BenchMark ULTRA, Ventana Medical Systems/Roche Diagnostics GmbH, USA). Merlin antibody was used at a dilution of 1:20 (NF2, B-12, sc-55575, Santa Cruz Biotechnology, USA). The OptiView DAB IHC Detection Kit (Roche Diagnostics) visualised the bound antibody. Hematoxylin served as counterstain. NF2-positive schwannoma tissue was employed as on-slide positive control.

## Results

### Meningioma cell culture characterisation

**Fig. 1:**
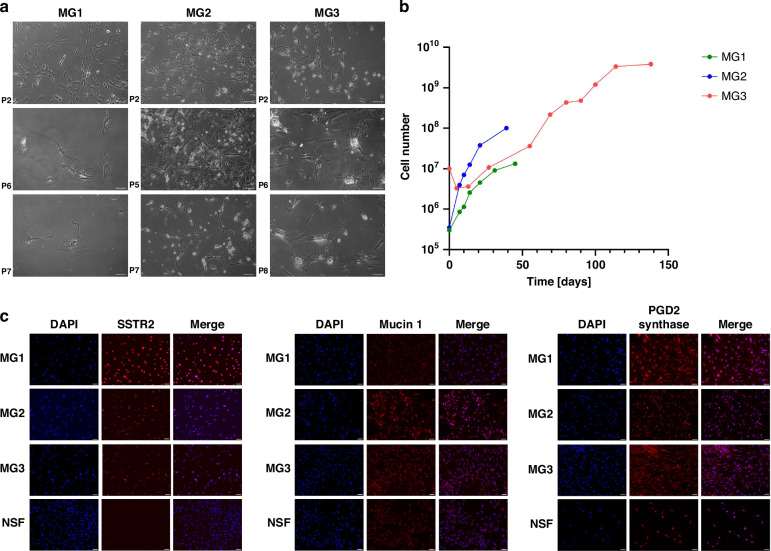
Characterisation of primary meningioma cell cultures MG1, MG2 and MG3. **a** Microscopic picture of the primary meningioma cell cultures at different passages. MG1, MG2 and MG3 are shown in a 10x magnification, scale = 20 µm. **b** Growth behaviour of the primary meningioma cell cultures. The dots indicate the passages. MG1 and MG2 start at passage 2, while MG3 was followed from passage 0. **c** Indirect immunofluorescence staining of SSTR2A, mucin 1 and PGD2 synthase; NSF nasal fibroblasts; scale bars = 50 µm.

The three primary meningioma cell cultures MG1, MG2 and MG3 were established from patient-derived tumour tissue of grades I or II (Supplementary Table S3). All three meningioma cell cultures showed unique cell morphologies. MG1 had a round tile-shaped morphology, MG2 cells were thin-shaped, and MG3 displayed a mixed thin as well as tile-shaped phenotype (Fig. 1a).

The meningioma cultures showed exponential growth between passages 2 and 6, while the proliferation decreased slightly in higher passages (Fig. 1b). MG2 showed the fastest growth with a doubling time of 3.11 days, followed by MG1 with 5.35 days and MG3 with 8.34 days (Supplementary Table S4). At the beginning of this growth phase, the doubling time was higher than at the end, with a doubling time of 4.66 days between passages 2 and 3 and 8.43 days between passages 5 and 6 for MG1, 2 days and 4.42 days respectively for MG2, and 2.56 days and 5.42 days respectively for MG3. After passage 6, the doubling time of all meningioma cultures increased strongly, with 10 days for MG1, 12.72 for MG2, and 11 for MG3. As cell proliferation speed decreased in higher passages, the range between passages 2 and 6 was chosen for pharmacological tests.

All primary cell cultures stained positive for the expression of meningioma markers SSTR2A, PGD2 synthase and Mucin 1 (Fig. 1c). SSTR2A was expressed most strikingly in all three primary meningioma cell cultures but not in the primary fibroblast control cells and is therefore suitable as a meningioma marker in our setting.

### Molecular targeting of meningioma cells

The most frequently inactivated tumour suppressor in meningiomas is Merlin, which coordinates membrane receptor signalling and cell-cell contact-mediated inhibition of the cell cycle [29]. As it plays a regulatory role in several well-established meningioma-driving signalling pathways, including those involved in cell survival, differentiation and proliferation [30, 31], we conducted pharmacological assays to evaluate small molecules and other drugs targeting these pathways in three meningioma cell cultures, using concentrations comparable to those in previous studies on other tumour cell types (Table 1).

**Table 1:** IC_50_ ranges of drugs targeting meningioma-driving signalling pathways tested on MG1, MG2 and MG3.

Drug	Target	IC_50_ range of MG1, MG2 and MG3	IC_50_ values from publications	Cell line type	Reference
Sulforaphane	NFκB NRF2 MAPK	34.4–40.1 µM	40 µM	Ovarian cancer	[57]
Metformin	mTOR, ERBB2, EGFR, AKT, STAT3, IGF-1	4.678–11.13 mM	12–14 mM	Breast cancer	[58]
Y15	FAK	2.908–5.025 µM	3–18 µM	Colon cancer, Thyroid papillary cancer	[59]
C188-9	STAT3	11.48–25.35 µM	3.7 µM	Head and neck squamous cell carcinoma	[60]
Imatinib Mesylate	PDGFR	10–100 µM	10–15 µM	Ovarian cancer	[61]
Delgocitinib	JAK	50–500 µM	2.6–58 nM	-	[62]
Trans-Zeatin	c-Jun, JNK	100–1000 µM	<166 µM	Leukaemia	[63]
AG-490	EGFR, JAK2, STAT3	50–500 µM	30.8 µM	Non-small cell lung cancer	[64]
Zibotentan	AKT, EGFR	50–50 µM/ > 500 µM	0.1–10 µM	-	[65]
Cetuximab	EGFR	>50 µM/ > 1000 µM	1–2 mg/ml (6.9–13.7 mM)	Non-small lung cancer	[66]

The drugs sulforaphane, metformin Y15, C188-9, imatinib, trans-zeatin, AG-490 and cetuximab were tested in different cancer types, and their effect on the cell viability was determined by their IC_50_ value. The drugs delgocitinib and zibotentan were not tested for their effect on the viability of cancer cells. The IC_50_ of delgocitinib describes its kinase inhibitory activity to the 4 JAKs JAK1, JAK2, JAK3 and the tyrosine kinase (TYK2) and the IC_50_ of zibotentan describes its receptor antagonist inhibition on the endothelin A receptor.

**Fig. 2:**
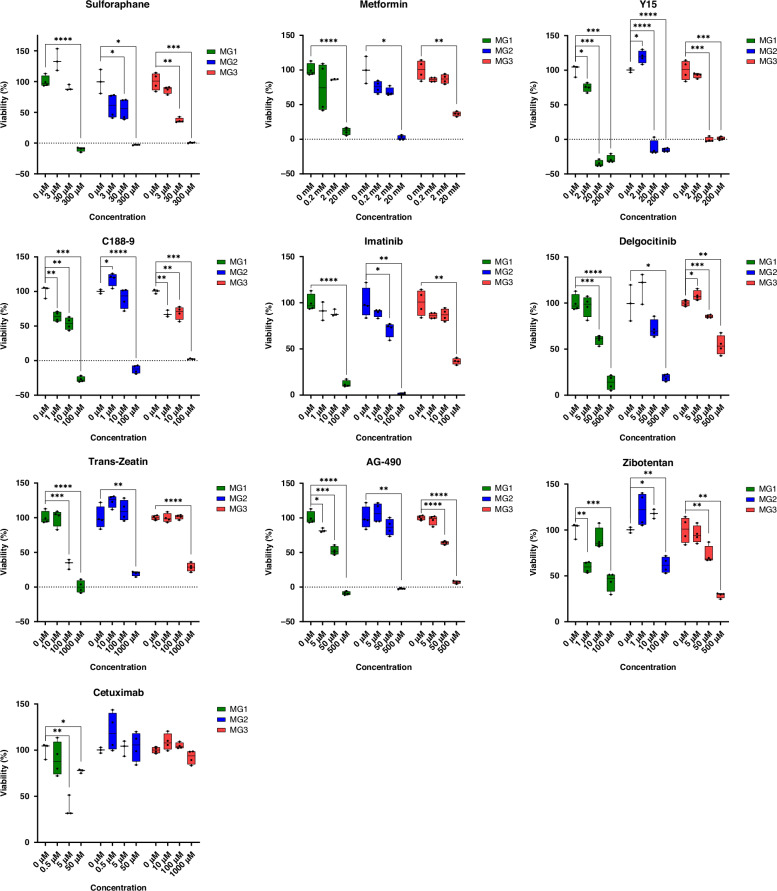
Drug screening in primary meningioma cell cultures MG1, MG2 and MG3. The drugs sulforaphane, metformin, Y15, C188-9, imatinib mesylate, delgocitinib, trans-zeatin, AG-490, zibotentan and cetuximab were tested in technical replicates (*n* = 4) to determine their effectiveness in all three cell cultures. The significance was calculated using an unpaired Student’s *t* test with Welch´s correction: *p < 0.05; **p < 0.01; ***p < 0.001; ****p < 0.0001.

Three drug concentrations were preliminarily assessed on primary cell cultures to determine the sensitivity of meningioma cells to these compounds. All tested drugs did affect the viability of the cells, at least in individual patients (Fig. 2 and Supplementary Table S5). Most prominently, antiphlogistic sulforaphane, type 2 diabetes medication metformin, focal adhesion kinase-inhibitor Y15, STAT3-inhibitor C188/9, PDGFR-inhibitor Imatinib and JAK-inhibitor Delgocitinib reduced cell viability in all three meningioma cultures.

The drugs sulforaphane, metformin, Y15 and C188-9 were chosen for further tests, as sulforaphane and metformin represent clinically approved drugs that could easily be repurposed for meningioma targeting. C188-9 and Y15 showed effectiveness at the lowest concentrations in our initial screening and thus were investigated further as well.

**Fig. 3:**
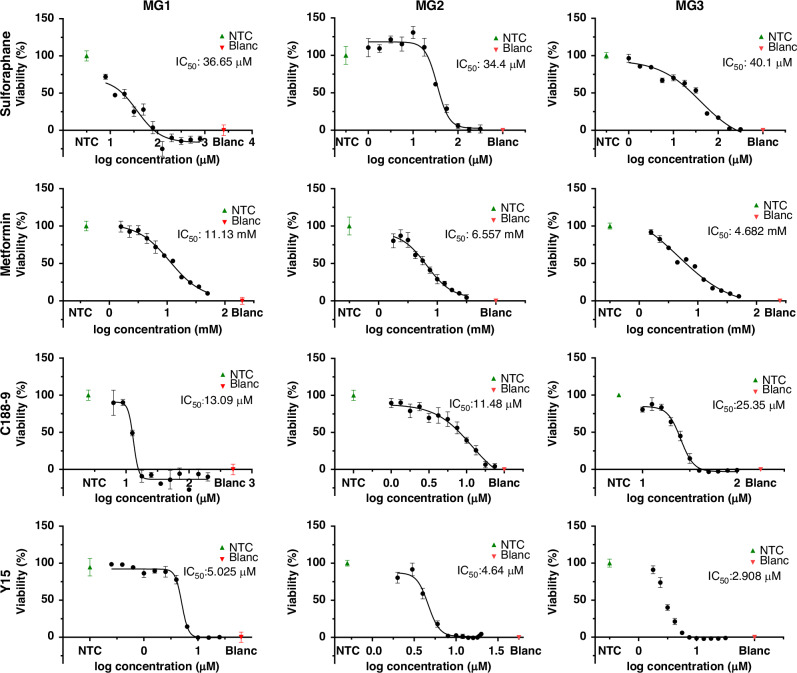
Single drug treatment in primary meningioma cell cultures. The primary cell cultures MG1, MG2 and MG3 were treated with a serial dilution of the drugs sulforaphane, metformin, C188-9 and Y15 separately using four technical replicates (*n* = 4). The effect of the drugs on cell viability was examined using an MTS assay. The data represent the mean and standard deviation.

Subsequently, the IC_50_ of these four drugs was determined using serial dilution experiments for MG1, MG2 and MG3 (Fig. 3 and Supplementary Table S6). The IC_50_ value of sulforaphane is 36.65 μM (error range: 12.9–52.72 μM), 34.4 μM (error range: 30.84–38.68 μM) and 40.1 μM (error range: 29.53–79.08 μM), respectively. The IC_50_ values for metformin are 11,130 μM (error range: 9489–13840 μM), 6557 μM (error range: 5488–7738 μM) and 4682 μM (error range: 8–6820 μM), for C188-9 13.09 μM (error range: 12.6–13.81 μM), 11.48 μM (error range: 8.929–40.2 μM) and 25.35 μM (error range: 24.43–26.28 μM) and for Y15 5.025 μM (error range: 4.792–5.265 μM), 4.64 μM (error range: 4.399–4.907 μM) and 2.908 μM (error range: 2.714–3.069 μM) for MG1, MG2 and MG3, respectively. The minimum efficiency dose (MED) of sulforaphane, metformin, C188-9 and Y15 was measured at 3–32 μM, 2–7 mM, 5–13 μM and 2–7 μM, respectively (Supplementary Table S7).

**Fig. 4:**
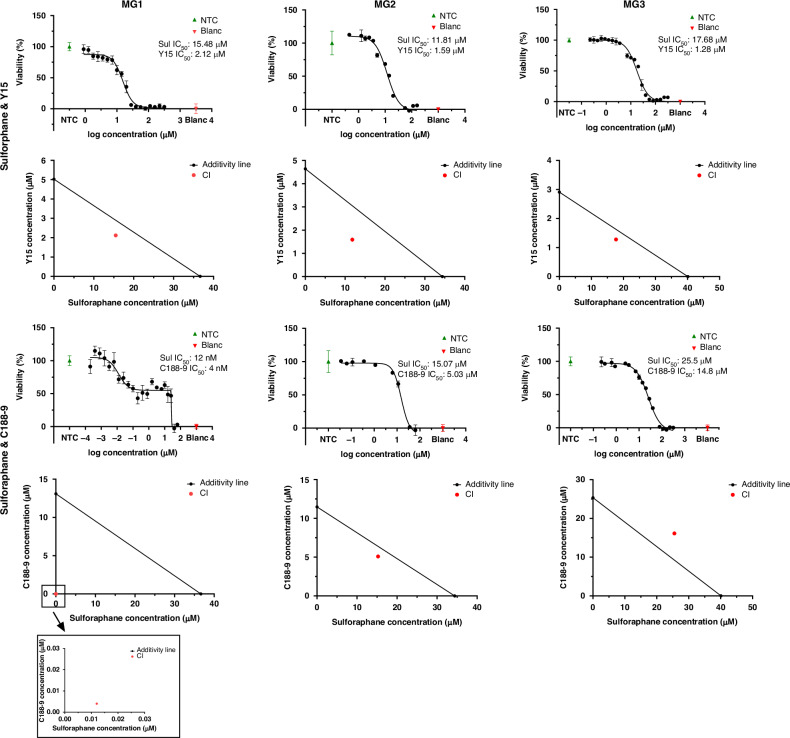
Drug-combination treatment and isobologram analysis of primary meningioma cell cultures. The primary cell cultures MG1, MG2 and MG3 were treated with a serial dilution of the drug combinations sulforaphane and Y15, as well as sulforaphane and C188-9 in four technical replicates (*n* = 4). The effect of the drugs on cell viability was measured using an MTS assay. Displayed is the log concentration of the drug sulforaphane. The data show the mean and standard deviation. The associated isobolograms display the combination index (CI) of the combined drugs as a red dot. A CI above the additivity line indicates an antagonistic effect, and a CI under the additivity line indicates a synergistic effect of the drugs.

Sulforaphane was combined with the other three drugs and tested in the meningioma cell cultures by performing a dilution series to determine the IC_50_ of the drugs in combination. The IC_50_ of all tested drugs was lower in the combination treatments. The IC_50_ value of sulforaphane decreased by 28.54% to 26.19 μM when combined with metformin, and it was lowered by 57.76% to 15.48 μM when used together with Y15. The IC_50_ of metformin decreased by 28.54% to 7953.47 μM in this experiment, and the IC_50_ of Y15 was measured 57.76% lower at 2.12 μM. Nonetheless, the combination of metformin and sulforaphane showed a clearly antagonistic effect in MG1, with a combination index (CI) of 1.43 (Supplementary Fig. S1) and therefore not tested further in MG2 and MG3. The combination of Y15 and sulforaphane was strikingly synergistic on all three meningioma cell cultures, while the combination of C188-9 and sulforaphane was tested synergistic in MG1 and MG2 but not in MG3, with CI values of 0.0007, 0.88 and 1.27, respectively (Fig. 4 and Supplementary Table S8). While the combination of sulforaphane with metformin and Y15 resulted in the typical S-shaped graph, the combination of sulforaphane and C188-9 showed two phases. The first represents the effect of low concentrations of the drugs on the cells at the range of about 10 nanomolar (nM) sulforaphane and 4 nM C188-9. At the range of 29 μM sulforaphane and 10 μM C188-9, a second decline of cell viability was detected, which we explain by unspecific chemical toxicity and which we thus excluded from the CI calculation.

As the combination of sulforaphane and Y15 was clearly the best meningioma-targeting approach in our system, we examined the impact of both drugs on cell biology. Indirect immunofluorescence (IF) staining revealed a strong loss of proliferation marker Ki67 staining in the nucleus of combination-treated cells compared to control cells (Supplementary Fig. S2). Simultaneously, IF revealed higher positivity of cleaved caspase 3 and apoptosis-inducing factor 1 (AIF) (Supplementary Fig. S3) in treated cells compared to untreated controls. This indicates a loss of proliferation and an induction of apoptosis in meningioma cells treated with sulforaphane and Y15.

As FAK inhibition was found synthetic lethal with Merlin deficiency in mesothelioma [32], we examined whether the observed drug synergy of sulforaphane and Y15 correlated with the *NF2* status in our meningioma model. Because positive Merlin immunohistochemistry was recently described as a surrogate marker for *NF2* mutation status in low-grade meningiomas [33], we analysed *NF2*/Merlin expression in the original tumour tissue. MG1 and MG2 stained highly positive, whereas MG3 was tested negative for Merlin expression, compared to the respective positive control (Supplementary Fig. S4). Thus, we conclude that the sulforaphane/Y15 drug action worked independently of the *NF2* status.

## Discussion

**Fig. 5:**
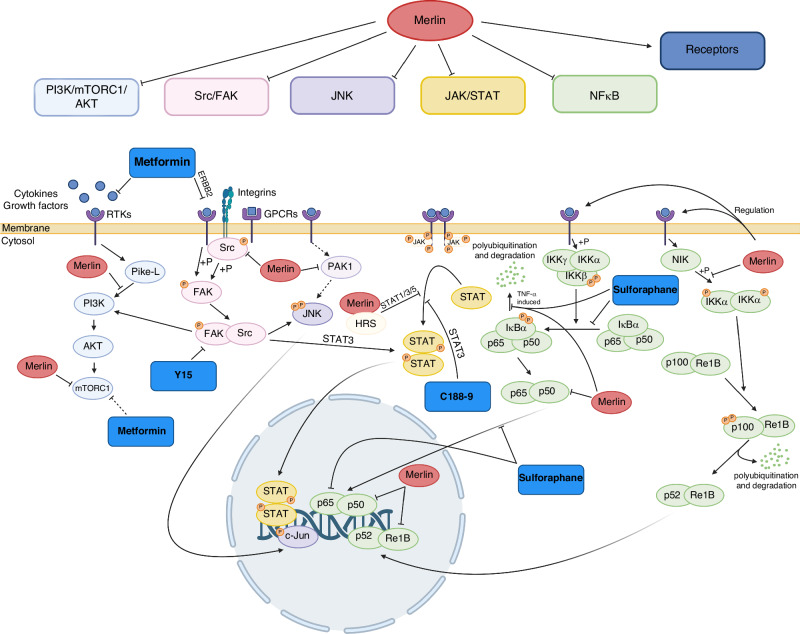
Inhibition of the meningioma signalling circuit. The PI3K/mTORC1/AKT-, the JNK-, the JAK/STAT-, and the NFκB pathway are suppressed by merlin [14–18]. Merlin also regulates cell surface receptors in their distribution and availability [53]. Mutations in the *NF2* gene lead to dysfunctional Merlin causing a deregulation of these pathways. Drugs targeting key signalling effectors were chosen to compromise cell viability. Metformin suppresses PI3K/mTORC1/AKT [42], FAK inhibition through Y15 targets PI3K/mTORC1/AKT, JNK and JAK/STAT [54], C188-9 inhibits JAK/STAT [55], and sulforaphane interferes with the NFκB pathway [56]. Created in BioRender. Schwab, M. C. (2026) https://BioRender.com/uomae6o

The data presented here indicate molecularly targetable vulnerabilities within the cell signalling programme of meningioma cells. So far, traditional chemotherapy is an exception in the clinical management of meningiomas and is rather used to treat faster-growing high-grade tumours, as their higher replication activity can be targeted by DNA-damaging agents. Molecular targeting approaches have been tested previously, and some have been translated into clinical trials, for example, targeting oestrogen receptor, receptor tyrosine kinases, and growth factor receptors [34]. Similar to our approach, mTOR inhibitor Everolimus has been clinically studied recently as treatment option for meningioma [35]. However, still no effective pharmacotherapy has been established for routine therapy. Thus, we tested compounds that aimed at the signalling pathway circuit that is implicated in the progression of this tumour disease and includes growth factor receptor signalling (inhibition of EGFR/PDGFR/JAK/STAT3/JNK/mTOR), inflammatory signalling (NRF2 activation, NFκB inhibition), and adhesion signalling (FAK inhibition) [31, 36, 37] (Fig. 5). However, there are more targets in the pathways downstream of Merlin, which are illustrated in Fig. 5, that remain unexplored and could be subject of future studies.

Strikingly, sulforaphane has a strong antioxidative and anti-inflammatory impact and targets meningioma cells in our system. This is especially valuable, as this drug is freely available as food supplement and could be repurposed for meningioma therapy immediately. In addition, FAK inhibition has previously been shown to be synthetic lethal with Merlin loss of function in malignant pleural mesothelioma [32] and it was investigated for meningioma therapy in a clinical trial [38]. However, our data rather indicate that the synergy between sulforaphane and FAK inhibition is not dependent on the *NF2* status.

Merlin regulates many tumour-promoting signalling pathways, including NFκB [16], which is inhibited by sulforaphane [23, 24]. Thus, suppressing inflammation appears to challenge meningioma cells and the expression of the pro-inflammatory effector COX-2 has been associated with an increasing meningioma grade [39]. Also, JAK/STAT signalling is activated by inflammatory cytokines and our data implicate STAT3 to be another possible target in meningioma cells, as cell viability was compromised upon inhibition of this molecule in our experimental system. JAK1/STAT3 signalling is negatively regulated by Merlin as well [17], and JAK1, p-JAK1, STAT3 and p-STAT3 are described to be highly expressed in grade I and II meningiomas and to drive VEGF expression, which is important for tumour progression [40]. Another interesting drug candidate in our set is metformin, which is a clinically well-established treatment for diabetes and showed meningioma cell-targeting activity in our single-drug tests. Accordingly, a lower risk of cerebral meningioma was shown in metformin-using diabetes patients [41]. Metformin can impact various pathways, including AKT/mTOR [42, 43], and it has already been shown to target meningiomas in synergy with cisplatin [44]. Here, we propose Metformin as a single compound treatment, which was not sufficiently investigated in single use previously, but intriguingly sulforaphane and metformin were found antagonistic in our study. The combination of both drugs was previously investigated in breast cancer revealing an additive effect with no synergy [45]. Even though we cannot explain this effect from our data, one reason for the incompatibility of metformin with sulforaphane may be the high concentration of metformin needed for its biological effect, which is one decimal power higher than the concentration of sulforaphane. This can cause differences in the death kinetics of cancer drugs, leading to antagonism [46]. This could be further examined in future studies.

In contrast, the combination of STAT3 inhibition and sulforaphane showed good results in two out of three patients and thus may be applicable in clinics, at least for certain patients. Moreover, sulforaphane targeted meningioma cells synergically with FAK inhibition in all three patients` primary cells. This indicates a potential pharmacotherapy approach against this tumour entity, which should be followed up in animal models and clinical trials. Moreover, our data suggests testing a larger set of compounds in combination with sulforaphane, expanding the set of usable combination partners. Particular emphasis should be placed on anti-inflammatory molecules, as our data suggest that inflammation plays a key role in the pathology of meningioma cells. This is further supported by the correlation between inflammatory marker expression and invasive growth as well as malignancy in this disease [39, 47].

Our study indicates a pharmacotherapy approach as the described compounds clearly compromise meningioma cell viability. However, future studies should be conducted to further evaluate the mode-of-action of the drugs and evaluate their effectivity in vivo, for example, using previously published mouse models [48]. Our results may also be relevant for other tumour types that show similar pathobiology, most prominently vestibular schwannomas [26].

The MED values for sulforaphane, metformin, C188-9, and Y15 observed in our study reside in the low micromolar range (except metformin), which indicates that plasma levels in that range would have to be reached in clinical use. Sulforaphane was used in a study of Heidelberg patients with pancreatic carcinoma. They were treated with 90 mg sulforaphane in addition to their chemotherapy and showed no severely impairing side effects [49, 50]. Ninety milligrams sulforaphane leads to the average sulforaphane plasma concentration of 13.34 µM [51], which is in range of the here measured MED for two out for three patients. As a significant difference can be achieved also by a lower concentration of sulforaphane than the calculated IC_50_, treating a patient with lower doses of sulforaphane over a longer period may influence meningiomas therapeutically. The average metformin blood concentration when taken 0.5–3 g for treating diabetes is about 1846 ng/ml (14 µM) [52]. The here observed MED range for metformin is a thousandth higher than that. Thus, the established metformin diabetes regimen may not be suitable for meningioma treatment, but alternative approaches using nanoparticles for example [45] may still be possible in future. C188-9 and Y15 are new compounds that must be further evaluated for their use in humans in future studies before their suitability for meningioma therapy can be estimated.

Altogether, the clinical application of oral drugs appears easier in meningiomas than in other brain tumours like gliomas, as they reside outside the brain-blood-barrier. Thus, we believe that molecular-targeted therapies will strongly benefit meningioma patients in future.

## Supplementary information

Supplementary Materials

## Data Availability

All materials are contained within the manuscript and the supplementary material.
