# Supplementary material for: Molecular targeting of the meningioma cell signaling circuit reveals drug vulnerabilities including synergy between sulforaphane and focal adhesion kinase inhibition
Source: BJC Rep. 2026 Apr 15;4:21. doi: 10.1038/s44276-026-00204-2 (PMC13083847; doi:10.1038/s44276-026-00204-2)
Supplement: Supplementary file 1 — Supplementary Materials [file 44276_2026_204_MOESM1_ESM.pdf]

Supplementary Materials

Supplementary Tables

Supplementary Table S1: Antibody list. The following primary and secondary antibodies were used in their described dilutions for indirect immunoflourescence staining of the primary meningioma cell cultures.

Antibody	Dilution	Manufacturer
Mouse-anti-human AIF mAb (B-9)	1:100	Santa Cruz Biotechnology, Dallas, TX, United States, Cat. No.: sc-55519
Rabbit-anti-human Cleaved Caspase (Asp175) (5A1E) mAb	1:200	Cell Signaling Technologies, Danvers, MA, United States, Cat. No.: 9664
Rabbit-anti-human Ki67 mAb (SP6)	1:200	Abcam, Cambridge, UK, Cat. No.: AB16667
Mouse-anti-human Mucin 1/MUC1 mAb (VU4H5)	1:100	Santa Cruz Biotechnology, Dallas, TX, United States, Cat. No.: sc-7313
Mouse-anti-human PGD2 synthase mAb (F-7)	1:100	Santa Cruz Biotechnology, Dallas, TX, United States, Cat. No.: sc-390717
Mouse-anti-human SSTR2 mAb (A-8)	1:100	Santa Cruz Biotechnology, Dallas, TX, United States, Cat. No.: sc-365502
Mouse-anti-human NF2 mAb (B-12)	1:20	Santa Cruz Biotechnology, Dallas, TX, United States, Cat. No.: sc-55575
Goat anti-Rabbit IgG (H+L) Cross-Adsorbed Secondary Antibody, Alexa Fluor™ 488	1:400	Thermo Fisher Scientific, Waltham, MA, United States, Ref.: A11008
Goat anti-Mouse IgG (H+L) Cross-Adsorbed Secondary Antibody, Alexa Fluor™ 555	1:400	Thermo Fisher Scientific, Waltham, MA, United States, Ref.: A-21422

Supplementary Table S2: Drug list. Drugs with their targets and clinical phase status used in pharmalogical tests on the primary meningioma cell cultures.

Drug	Target	Clinical trial phase	Manufacturer	Formula*
Sulforaphane	NFκB NRF2 MAPK	4	Sellekchem, Germany, Cat No: S5771	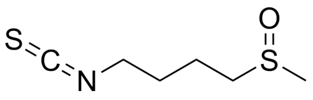
Metformin	mTOR, ERBB2, EGFR, AKT, STAT3, IGF1	4	Sellekchem, Germany, Cat No: S5958	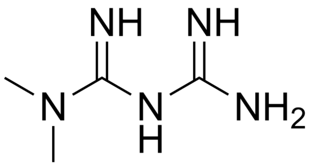

C188-9	STAT3	2	MedChemExpress, Germany, Art.-Nr.: HY-112288	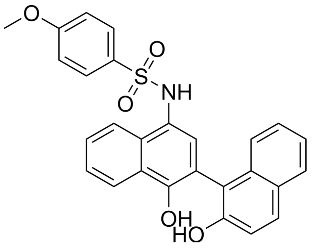
Y15	FAK	2	MedChemExpress, Germany, Art.-Nr.: HY-12444	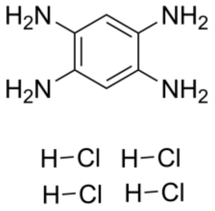
Imatinib mesylate	PDGFR	4	Selleckchem, Germany, Cat No: S1026	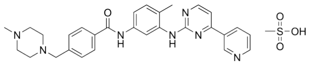
Delgocitinib	JAK	3	Selleckchem, Germany, Cat No: S8802	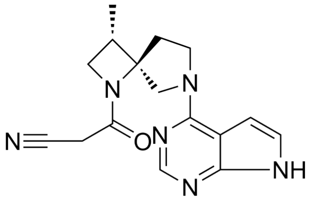
Trans-Zeatin	c-Jun, JNK	No record	Selleckchem, Germany, Cat No: S4884	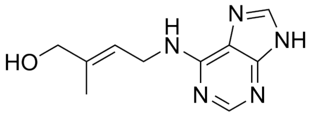
AG-490	EGFR, JAK2, STAT3	No record	MedChemExpress, Germany, Art.-Nr.: HY-120000	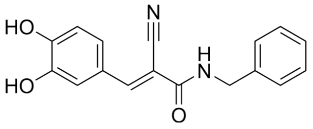
Zibotentan	AKT, EGFR	3	Selleckchem, Germany, Cat No: S1456	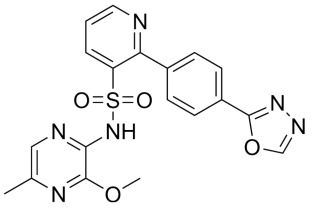
Cetuximab	EGFR	4	Selleckchem, Germany, Cat No: A2000	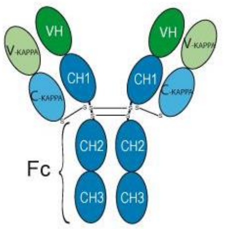

* Formulas taken from www.medchemexpress.com

Supplementary Table S3: Patient characteristics.

Patient	Age	Sex	Subtype	WHO grade
MG1	70	female	Transitional	I
MG2	68	male	Atypical meningothelial	II
MG3	53	female	Angiomatous	I

Supplementary Table S4: **Doubling time of primary meningioma cell cultures MG1, MG2 and MG3 in different passages.** The doubling time of the cells determined for the exponential growth phase and between the passages of that range.

Time range	MG1 [days]	MG2 [days]	MG3 [days]
Passage 2-6	5.36	3.11	8.34
Passage 2-3	4.66	2	2.56
Passage 3-4	7.23	3.63	8.83
Passage 4-5	3.42	4.78	16.1
Passage 5-6	8.43	4.42	5.42
Passage 6-7	10	12.72	11

Supplementary Table S5: Drug prescreening results. Shown are the median, mean and standard deviation (SD) of the viability of the primary meningioma cell cultures MG1, MG2 and MG3 after exposure to the chosen drugs at different concentrations. Zibotentan was used in concentrations of 1 µM, 10 µM and 100 µM on MG1 and MG2 and in concentrations of 5 µM, 50 µM and 500 µM on MG3. Cetuximab was used in concentrations of 0.5 µM, 5µM and 50 µM on MG1 and MG2 and in concentrations of 10 µM, 100 µM and 1000 µM on MG3.

Drug	Conc.	MG1 Viability Median/Mean/SD			MG2 Viability Median/Mean/SD			MG3 Viability Median/Mean/SD		
Sulforaphane	0 µM	96.83	100	7.79	99.66	100	15.95	100.88	100	12.10
	3 µM	132.53	134.73	14.49	61.59	60.57	17.10	88.57	86.67	4.99
	30 µM	87.59	89.89	3.84	55.98	55.22	14.93	35.39	36.88	3.41
	300 µM	-8.61	-9.86	3.00	-2.63	-2.80	0.51	0.73	0.66	0.70
Metformin	0 mM	96.83	100.00	7.79	99.66	100.00	15.95	100.88	100.00	12.10
	0.2 mM	74.36	134.73	14.49	75.86	75.43	7.54	86.13	86.06	2.91
	2 mM	86.77	89.89	3.85	66.17	68.38	5.18	86.94	86.94	5.78
	20 mM	12.20	-9.86	3.00	1.44	2.12	2.88	36.75	36.61	2.88
Y15	0 µM	104.61	100.00	7.22	100.23	100.00	2.50	100.88	100.00	12.10
	2 µM	75.49	75.00	5.27	119.21	119.21	7.81	93.44	92.42	3.05
	20 µM	-36.51	-35.03	4.04	-17.83	-12.92	9.36	-1.84	-0.48	3.11
	200 µM	-31.09	-28.87	4.85	-15.80	-15.46	2.42	1.14	1.35	1.99
C188-9	0 µM	104.61	100.00	7.22	100.23	100.00	2.50	100.71	100.00	2.42
	1 µM	63.65	63.65	6.10	120.57	117.86	8.15	66.58	67.70	3.94
	10 µM	53.78	53.54	7.66	93.44	90.22	12.64	71.00	69.10	8.04
	100 µM	-28.13	-27.14	3.45	-12.07	-12.58	5.08	1.98	2.21	0.59
Imatinib	0 µM	96.83	100.00	7.79	97.25	100.00	13.90	100.88	100.00	12.10
	1 µM	91.18	90.99	8.10	90.90	89.21	3.65	86.13	86.06	2.91
	10 µM	87.32	88.88	3.02	73.97	71.12	6.92	86.94	86.94	5.78
	100 µM	10.68	11.99	3.14	1.18	1.40	0.79	36.75	36.61	2.88

Delgocitinib	0 µM	96.83	100.00	7.79	99.66	100.00	15.95	100.10	100.00	2.84
	5 µM	98.76	96.55	9.74	122.43	117.33	13.66	107.14	108.26	4.61
	50 µM	61.27	60.03	4.28	69.59	72.05	8.41	85.32	85.52	1.47
	500 µM	14.13	13.71	6.77	19.81	19.30	3.26	53.29	54.26	9.03
Trans-zeatin	0 µM	96.83	100.00	7.79	97.25	100.00	13.90	100.10	100.00	2.84
	10 µM	104.27	100.07	10.28	126.24	123.80	7.55	98.47	99.64	5.58
	100 µM	34.94	33.29	5.75	108.68	110.26	12.89	101.94	101.12	2.76
	1000 µM	-0.21	0.55	7.60	20.44	19.38	2.91	28.82	28.77	5.30
AG-490	0 µM	96.83	100.00	7.79	97.25	100.00	13.90	100.10	100.00	2.84
	5 µM	81.25	82.08	2.43	105.92	107.09	12.28	98.98	96.84	6.01
	50 µM	51.62	52.65	5.28	86.46	86.67	10.23	64.10	63.95	2.04
	500 µM	-9.03	-8.82	2.21	-2.45	-2.35	0.76	7.40	7.10	1.72
Zibotentan	0 µM	104.61	100.00	7.22	100.23	100.00	2.50	100.88	100.00	12.10
	1 µM/ 5 µM	59.70	59.46	5.24	121.93	122.26	15.73	93.98	95.20	8.13
	10 µM/ 50 µM	85.36	90.05	10.26	117.86	117.63	4.16	68.41	72.67	8.22
	100 µM/ 500 µM	47.37	43.91	8.87	61.57	61.91	7.51	29.98	28.83	2.49
Cetuximab	0 µM	104.61	100.00	7.22	100.23	100.00	2.50	100.10	100.00	2.84
	0.5 µM/ 10 µM	87.83	90.30	15.87	117.86	119.72	17.91	107.85	108.87	7.73
	5 µM/ 100 µM	31.58	38.16	9.30	104.29	102.49	6.77	103.67	104.69	2.80
	50 µM/ 1000 µM	77.96	77.30	1.68	105.65	103.79	13.71	93.78	92.35	6.49

Supplementary Table S6: IC_{50} of the drugs sulforaphane, metformin, C188-9, and Y15 targeting primary meningioma cell cultures.

Cell culture	Drug	IC_{50} [µM]	Error range [µM]	R squared
MG1	Sulforaphane	36.65	12.9-52.72	0.9023
	Metformin	11130	9489-13840	0.9744
	C188-9	13.09	12.61-13.81	0.9514
	Y15	5.025	4.792-5.265	0.9882
MG2	Sulforaphane	34.4	30.84-38.68	0.9682
	Metformin	6557	5488-7738	0.9551
	C188-9	11.48	8.929-40.2	0.9424
	Y15	4.64	4.399-4.907	0.9731
MG3	Sulforaphane	40.1	29.53-79.08	0.9664
	Metformin	4682	8-6820	0.9773
	C188-9	25.35	24.43-26.28	0.9831
	Y15	2.908	2.714-3.069	0.9915

Supplementary Table S7: Minimum efficiency dosis (MED) of sulforphane, metformin, C188-9 and Y15. The significance was calculated with a One-way Brown-Forsythe and Welch ANOVA test with a Dunnett's T3 multiple comparisons test in GraphPad Prism10.

	MG1		MG2		MG3	
	**MED**	**Adj. P-value**	**MED**	**Adj. P-value**	**MED**	**Adj. P-value**
**Sulforaphane**	7.94 µM	0.0123	31.62 µM	0.0414	3.16 µM	0.0455
**Metformin**	6.3 mM	0.0288	4.22 mM	0.0243	2.24 mM	0.0227
**C188-9**	12.59 µM	0.0004	5.62 µM	0.0245	10 µM	0.0027
**Y15**	6.31 µM	0.0049	2 µM	0.0365	2.37 µM	0.0074

Supplementary Table S8: IC_{50} values and combination indexes of the drug combinations sulforphane & Y15 and sulforphane & C188-9 in primary meningioma cell cultures.

Cell culture	Combination	IC_{50} [µM]	Error range [µM]	R squared	CI	Error Range
**MG1**	Sulforaphane & Y15	15.48<br>2.12	14.17-16.82<br>1.94-2.31	0.9721	0.84	0.77-0.92
	Sulforaphane & C188-9	1. 0.012<br>1. 0.004	0.008-0.019<br>0.003-0.007	0.8904	0.0007	0.0004-0.006
**MG2**	Sulforaphane & Y15	11.81<br>1.59	10.33-13.41<br>1.39-1.8	0.9859	0.69	0.6-0.78
	Sulforaphane & C188-9	15.07<br>5.03	13.07-18.18<br>4.36-6.06	0.9906	0.88	0.76-1.06
**MG3**	Sulforaphane & Y15	17.68<br>1.28	15.64-19.82<br>1.13-1.43	0.9802	0.88	0.78-0.99
	Sulforaphane & C188-9	25.5<br>14.8	23.42-27.83<br>14.8-17.59	0.9913	1.27	1.17-1.39

Supplementary Figures

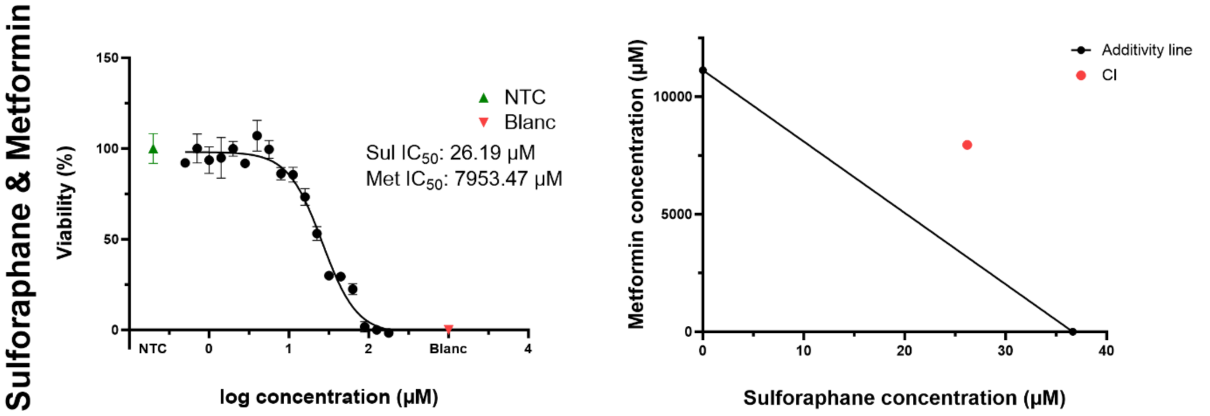

Supplementary Figure S1: Drug-combination treatment and isobologram analysis of sulforaphane and metformin in meningioma cells. The primary cell culture MG1 was treated with a serial dilution of the drug combination sulforaphane and metformin in four technical replicates (n=4). The effect of the drugs on the cell viability was measured using an MTS assay. Displayed is the log concentration of the drug sulforaphane. The data represent the mean and standard deviation. The associated isobologram shows the combination index (CI) of the combined drugs as a red point. A CI above the additivity line indicates an antagonistic effect.

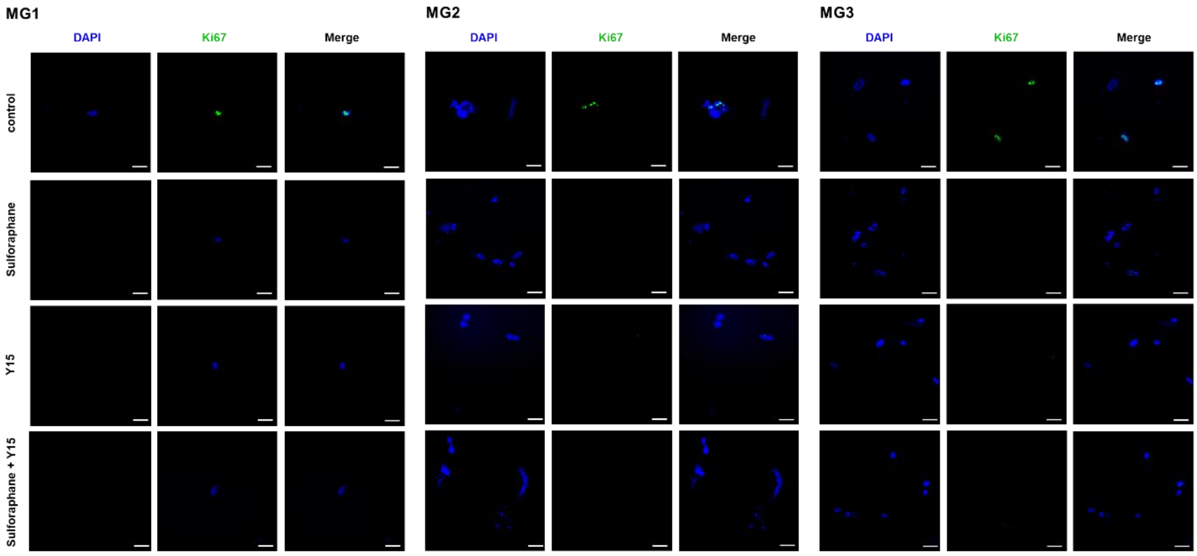

Supplementary Figure S2: Sulforaphane and FAK inhibition cause loss of proliferation marker Ki67. MG2 and MG3 primary meningioma cell cultures were treated with sulforaphane and/or FAK inhibitor Y15 for 24 hours. PFA fixated were subsequently stained for Ki67; DNA was stained using DAPI; drug concentrations were set at 1/2 of the respective IC_{50} in combination treatment experiments. Scale bars = 10µm.

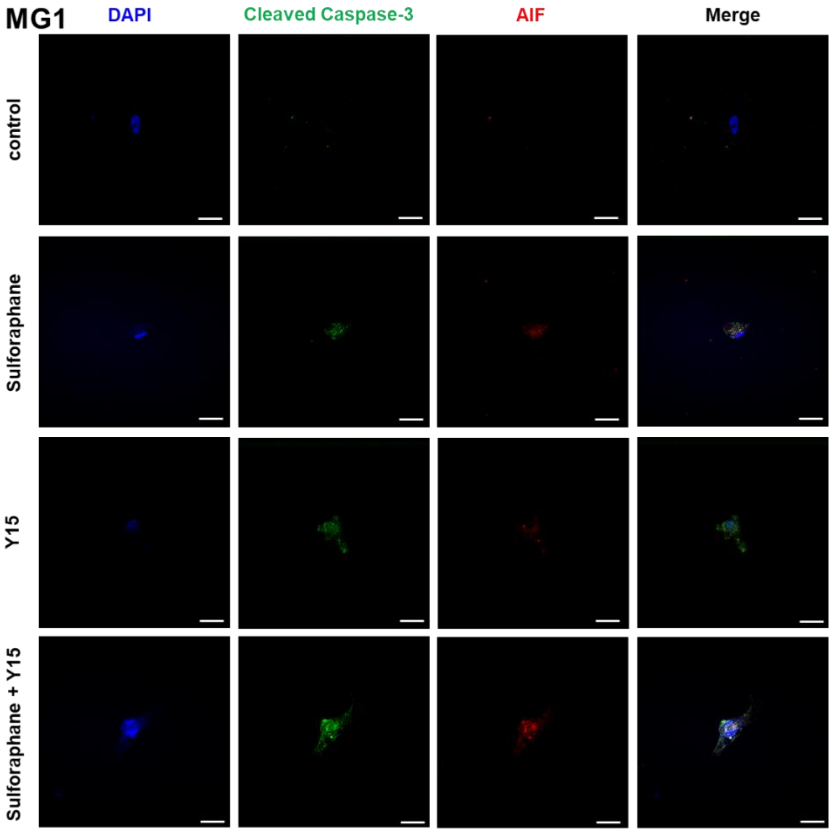

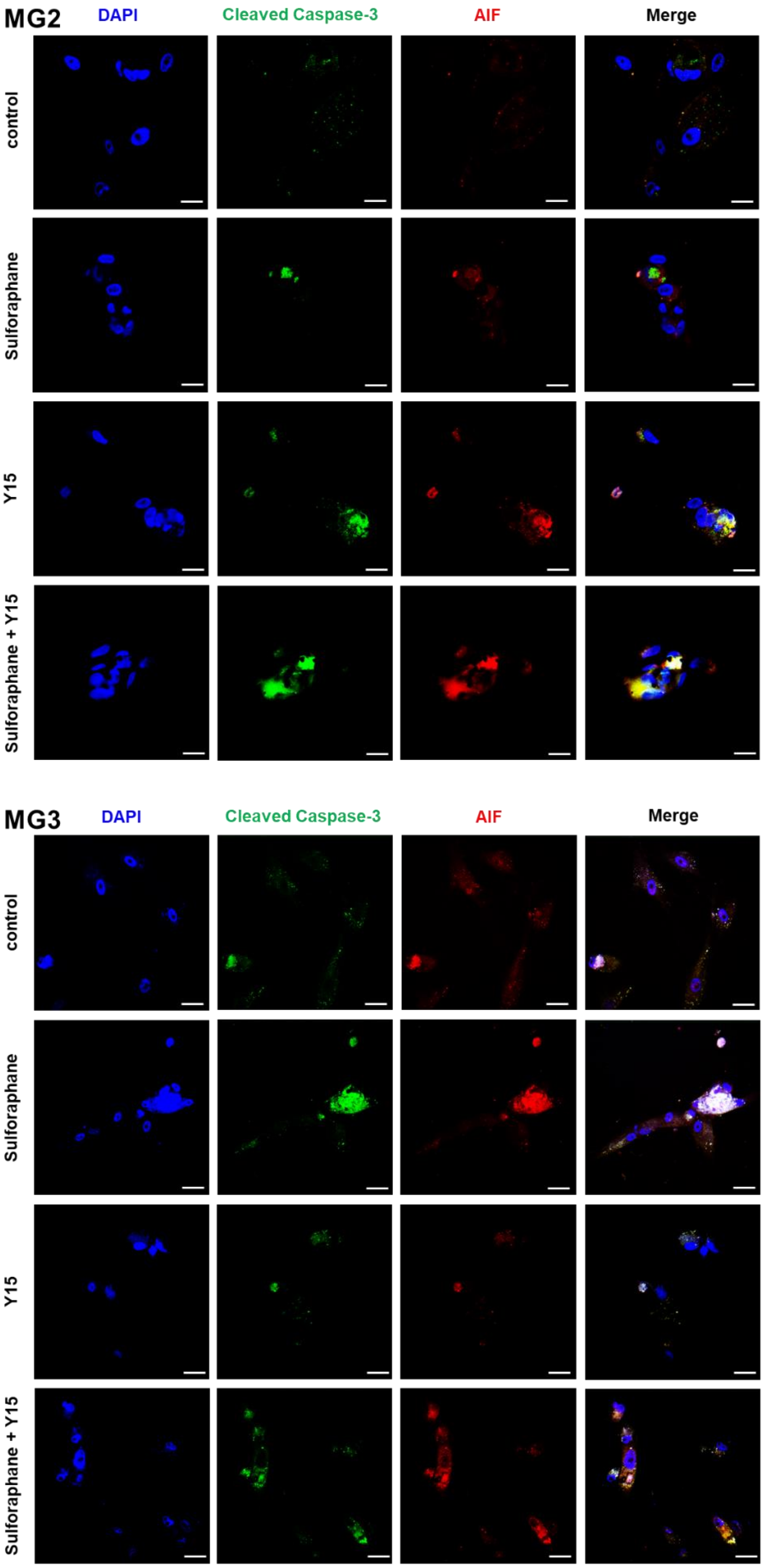

Supplementary Figure S3: Sulforaphane and FAK inhibition cause upregulation of apoptosis markers. MG1, MG2, and MG3 primary meningioma cell cultures were treated with sulforaphane and/or FAK inhibitor Y15 for 24 hours and stained for cleaved caspase 3 and apoptosis inducing factor (AIF); DNA was stained using DAPI; drug concentrations were set at 1/2 of the respective IC_{50} in combination treatment experiments. Scale bars = 10µm.

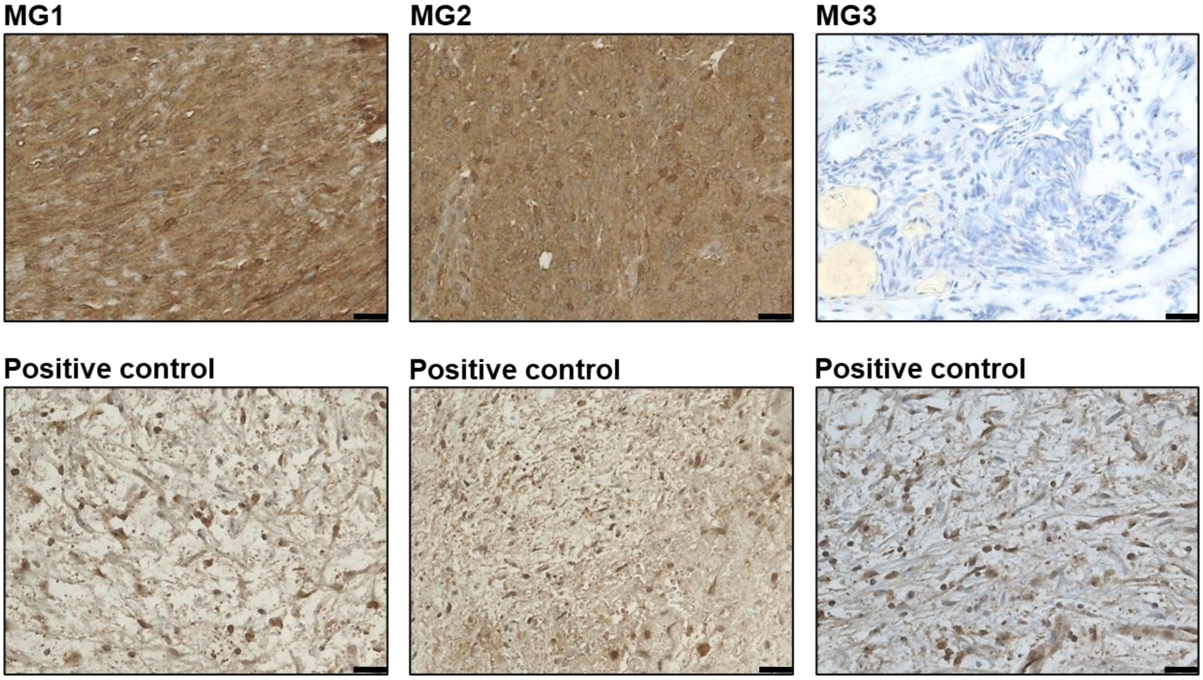

Supplementary Figure S4: Histopathology analysis of Merlin (*NF2*) expression in meningioma tissue. Tissue slides derived from the original tumors of MG1, MG2, and MG3 as well as on-slide positive controls were stained for Merlin protein using standard procedures. Representative images are shown. MG1 and MG2 show a positive staining for Merlin, whereas MG3 tumor tissue was tested negative. Scale bars = 20µm.
